# African Swine Fever in a Bulgarian Backyard Farm—A Case Report

**DOI:** 10.3390/vetsci6040094

**Published:** 2019-11-21

**Authors:** Laura Zani, Klaas Dietze, Zlatina Dimova, Jan Hendrik Forth, Daniel Denev, Klaus Depner, Tsviatko Alexandrov

**Affiliations:** 1 Friedrich-Loeffler-Institut, Südufer 10, 17493 Greifswald-Insel Riems, Germany; Klaas.Dietze@fli.de (K.D.); Janhendrik.Forth@fli.de (J.H.F.); Klaus.Depner@fli.de (K.D.); 2 Regional Food Safety Directorate Varna, 9000 Varna, Bulgaria; dr.zdimova@gmail.com; 3 Bulgarian Food Safety Agency, 1606 Sofia, Bulgaria; d_denev@bfsa.bg (D.D.); T_Alexandrov@bfsa.bg (T.A.)

**Keywords:** African swine fever, backyard sector, case report, ASFV whole-genome sequencing

## Abstract

African swine fever (ASF) is one of the most threatening diseases for the pig farming sector worldwide. As an effective vaccine is lacking, strict application of control measures is the only way to fight the disease in both industrial farms and backyard holdings. With generally low biosecurity standards, the latter are at particular risk for disease introduction and offer challenging conditions for disease control. In the following case report, we describe the overall course of an ASF outbreak in a Bulgarian backyard farm and the implemented control measures. Farm facilities and available data have been investigated to estimate the possible source, spread and time point of virus introduction. Contact with contaminated fomites entering the stable via human activities was regarded to be the most likely introduction route. The slow disease spread within the farm contributes to the hypothesis of a moderate contagiosity. As no further ASF outbreaks have been detected in domestic pig farms in the region, it could be demonstrated that successful disease control in small-scale farms can be reached. Thus, the report contributes to a better understanding of ASF in the backyard sector.

## 1. Introduction

**Figure 1:**
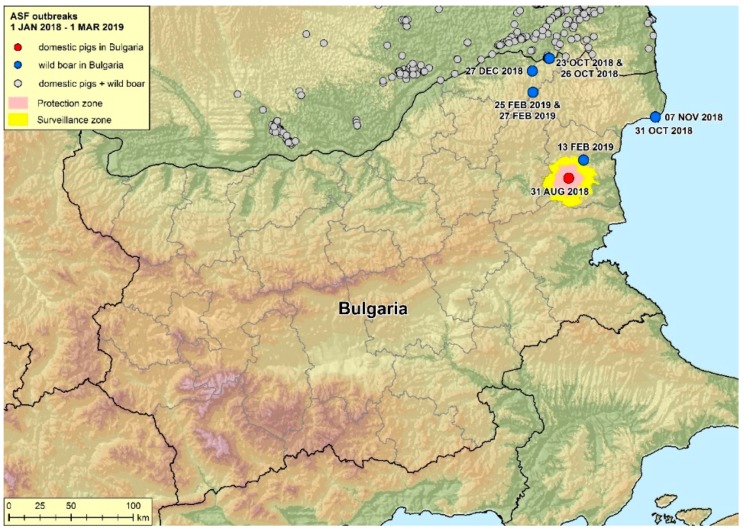
Map of reported ASF outbreaks in Bulgaria and bordering areas (1 January 2018–1 March 2019). The red dot marks the location of the described Bulgarian backyard farm. Blue dots indicate Bulgarian wild boar cases. ASF cases in bordering countries are colored in grey. Protection and surveillance zones are mapped according the COMMISSION IMPLEMENTING DECISION (EU) 2018/1280. P. Wysocki, Friedrich-Loeffler-Institut, Source: ADNS.

African swine fever (ASF), a fatal disease in domestic pigs and wild boar, has become a serious economic threat to the pig farming sector with global ramifications, more than ever underlined by the recent spread to China, Mongolia and Southeast Asia [1]. It is causing tremendous losses in the industrial pig sector but also affecting livelihood of small-scale pig holders in rural areas. In 1921, the disease was first described in Africa, where the disease is endemic. Nowadays it occurs in most countries with notable pig sectors [2,3]. The current Eurasian epidemic started in Georgia in 2007 [4] from where it spread through the Caucasus, Russian Federation, Ukraine and Belarus. In 2014, ASF arrived in the Baltic States and Poland where it became endemic in the wild boar population. In the following years, the disease spread to the Czech Republic, Hungary, Romania and Belgium. In Romania, hundreds of backyard outbreaks occurred in the south towards the Bulgarian border in 2018 (Figure 1). Against this background, Bulgaria was and still is under high risk of disease introduction. 

Backyard farms with their often-low biosecurity standards are considered particularly prone to disease introduction and thus are of particular interest in disease prevention and control. However, small-scale farming is common in rural areas in many countries of the world and still represents a significant part of agricultural practices [5]. It serves as an important or even the only source of meat supply for the population and often generates valuable cash income. In Bulgaria 96% of all pig holdings are classified as backyard farms (not more than 10 pigs) [6] containing 34% of the total pig population [7]. In August 2018, ASF was confirmed in a Bulgarian backyard farm with seven pigs in the region of Varna. It was the first occurrence of the disease in the country. 

In this case report we describe the overall course of the disease in that backyard and implemented control measures. We investigated available data to estimate the possible time point of disease introduction and to assess the high-risk period, as well as potential infection sources. From the African continent publications are available addressing ASF outbreaks in medium-sized farms [8] and the backyard sector [9]. Within the European context, there are only few case reports published [10], concerning mainly industrial pig farms [11] while for the backyard setting, reports are still lacking [12]. Hence, this case report contributes to a better understanding of outbreak pattern of ASF in small-scale holdings. In regard to the high number of outbreaks in Romanian backyard farms and the spread of the disease in China where 60% of pigs are kept in small-scale pig holdings [1] this knowledge could support effective disease control beyond the Bulgarian context. 

## 2. Methodology

Outbreak investigations have been performed by local veterinary authorities as required by EU legislation [13]. For detailed investigation of the outbreak, farm facilities have been inspected and information from the farmer and other stakeholders have been collected by semi-structured interviews. Main topics were farm settings, disease timeline on the farm, clinical and post-mortem findings, biosecurity conditions, animal movements, persons with recent access to the farm and feed purchase. Furthermore, laboratory results of diagnostic samples have been taken into consideration. In consequence, different hypothetical introduction pathways have been evaluated according to their probability. The hypotheses-based approach has been already described in a case report by Lamberga, Seržants and Oļševskis [11]. For the estimation of the high-risk period mortality data, laboratory results and clinical findings were analyzed as published by Nurmoja, et al. [14]. Thus, an incubation period of ~5 days was assumed [15,16]. The average survival time after the infection with an ASFV Genotype II was assumed to be 10 days [17].

Blood donor pigs kept for laboratory investigation at Friedrich-Loeffler-Institut, Germany were approved by the competent authority (Landesamt für Landwirtschaft, Lebensmittelsicherheit und Fischerei (LALLF) Mecklenburg-Vorpommern) under reference number LALLF 7221.3-2-041/17. No further animal experiments have been conducted within the present study.

## 3. Case Description 

### 3.1. Settings

**Figure 2:**
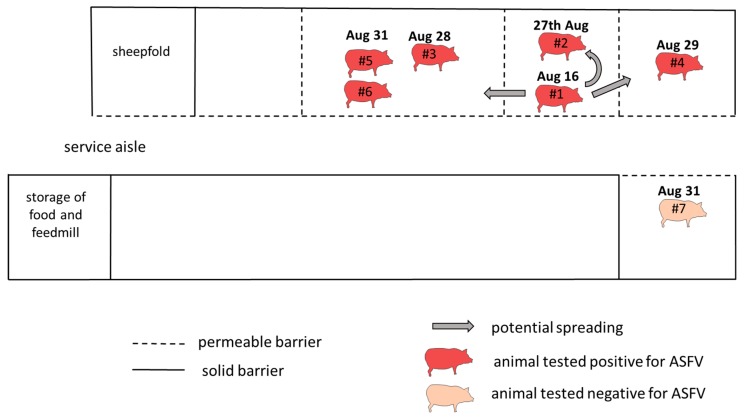
Potential spreading scenario.

The affected holding was located in a small village (120 households) in the rural area of the Varna region. Approximately half of the households in the village kept pigs under backyard conditions. The landscape is dominated by agricultural fields on the plains and patches of forest in the hilly areas. Backyard holdings of pigs and other livestock are very common as they are part of the traditional self-sustaining agriculture. In the affected farm, seven pigs (2 sows, 4 fattening pigs, 1 boar) were kept along with sheep, turkey, rabbits and chicken (see Figure 2). The pigs were housed in pens of ~six square meters. Half of the pen was an enclosed outdoor area where the pigs had direct contact with each other through fence bars while the other half was a closed housing. Water was supplied by hoses in each pen coming from the local fresh water pipeline. Pigs were fed with self-mixed compound feed produced from locally produced crops (mainly sunflower seeds and wheat) provided by the local agricultural cooperative. According to the farmer, no swill was fed to the pigs. He and his family were mainly taking care of the animals. In case of absences, colleagues or neighbors helped. Stable facilities were located on the enclosed premises surrounded by a stone wall. There was no demarcation for people visiting the farmer’s household between the recreational garden and the animal facilities. All pigs were purchased in 2017, when the farmer decided to restart pig breeding. The boar was occasionally brought to neighboring farms for breeding purposes. According to the farmer, the last movement was in May 2018. 

The potential spreading scenario has been reconstructed according to the chronological sequence of disease events and the interpretation of laboratory results. Animals are numbered consecutively according to their time point of death.

### 3.2. Timeline of the Disease Event

**Figure 3:**
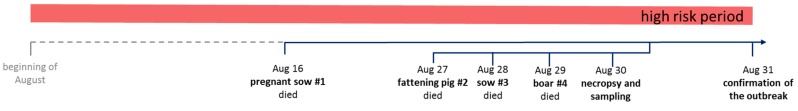
Timeline of the outbreak and estimated high-risk period.

On 16 August, one pregnant sow (#1) was found dead after it had been medically treated by the veterinary technician due to unspecific clinical signs (fever, lethargy and reduced feed intake) shown the days before. As the farmer and his family were absent, a colleague buried the carcass. On 27 August, the first fattening pig (#2) died, after being treated medically (antibiotics, antiphlogistics) because of showing similar unspecific clinical signs like reported from sow #1. The next day (28 August), the second sow (#3) died under comparable circumstances and was buried by the farmer together with the carcass of the fattening pig. On 29 August, the boar (#4) died and the farmer informed the mayor of the village (see Figure 3) who notified the competent veterinary authorities. Necropsy was performed, revealing findings indicative for ASF such as hemorrhagic lymph nodes, splenomegaly and petechial bleeding in the renal cortex. Samples were tested at the Bulgarian National Reference Laboratory in Sofia and have been retested by the European Union Reference Laboratory for African swine fever in Madrid (quantitative polymerase chain reaction (qPCR), immunoperoxidase test, virus isolation). Spleen, kidney and lymph node tissues of the boar (#4) were tested positive for ASFV specific antibodies by immunoperoxidase test and African swine fever virus (ASFV) genome by qPCR. Furthermore, ASFV could be isolated from spleen and lymph nodes. After the official confirmation on 31 August, the remaining three pigs (#5-7) were culled and tested. At that time, two fatteners kept in the same pen showed unspecific clinical signs like lethargy, anorexia and fever up to 41°C and were tested positive for ASFV (virus isolation and viral genome). One of them (#5) was also positive for ASFV specific antibodies (low titer; 1:20) while the other pig (#6) was tested antibody negative. The third fattener (#7), kept in a separate pen across the service aisle, did not show any signs of disease and was tested negative for both ASFV specific antibodies and ASFV genome. The two carcasses that were buried before the confirmation of the ASF outbreak, have been excavated and sampled. Both carcasses were tested positive for ASFV genome (see Table 1).

### 3.3. Control Measures 

Control measures were performed according EU legislation. All domestic pigs of the village, 90 adult pigs plus the offspring of one sow, exclusively placed in backyard holdings, were culled until 3 September. Since none of the pig farms in the village was officially registered, Bulgarian compensation policy did not apply. The culled animals were buried in a pit in vicinity to the village as recommended by the Bulgarian Ministry of Environment. Stables and potentially contaminated roads were cleaned and disinfected by competent authorities. The same applied for all vehicles and instruments included in the culling procedure. The three carcasses improperly disposed of by the farmer before disease confirmation have been sampled and buried at 2 m depth, together with calcium hydroxide (lime). The ground surface and all involved vehicles were sprayed with Virkon^®^ S (Lanxess) afterwards. In order to ensure that the disease did not spread to other villages, samples have been taken from pigs in the 10-km restriction zone around the outbreak farm. Between 3 September and 18 September, 90% of the domestic pigs (70 in total) kept in the restriction zone were sampled and clinically assessed. All samples were tested negative for ASFV by qPCR and ELISA. Farmers, keeping pigs in traditional free-ranging herds, e.g., East Balkan Swine [18], were obliged to keep them temporarily in enclosed areas. All pig owners were reminded that it is mandatory to register holdings and apply basic biosecurity measures. Farmers in restriction zones were obliged to immediately report dead pigs to the authorities for ASF testing. Hunters were obliged to sample shot wild boar within the restriction zone and to report and sample found dead wild boar. It is not allowed to move or sell meat until the hunted wild boar has been tested negative for ASF.

### 3.4. Genetic Analysis

Whole genome sequencing was performed from spleen tissue of pig #4 that succumbed to ASF during the outbreak. The applied protocol has been described elsewhere [19]. The resulting whole genome sequence of ASFV-Bulgaria 2018/1 (available from the European Nucleotide Archive under study accession number PRJEB35228) showed a mean coverage of 224 and a length of 190.587 bp. When compared to other ASFV-sequences, we identified an overall sequence identity of more than 99.9% to sequences from Eastern Europe (ASFV Moldova 2017/1, LR722599.1; ASFV Czech Republic 2017/1, LR722600.1), Western Europe (ASFV Belgium 2018/1, LR536725.1) and Asia (ASFV China/2018/AnhuiXCGQ, MK128995). Therefore, the available whole-genome sequence information does not lead to any further conclusions on the origin of ASFV Bulgaria 2018/1.

## 4. Discussion

### 4.1. High-Risk Period

The high-risk period (HRP) is regarded as the length of time that ASFV may have existed on a farm before the disease is suspected [14]. Estimation of this period is legally required for effective tracing back of possible disease spread [13]. For this purpose, laboratory findings and mortality data were taken into consideration as mentioned above. The first pig succumbed to ASF infection on 16 August. With an assumed survival time of around 10 days post infection, the most likely time of disease introduction was the beginning of August 2018. Thus, the time between virus introduction and ASF suspicion would be around 25 days (see Figure 3). This is longer compared to the median HRP (11 days) observed in domestic pig outbreaks in Estonia [14]. The longer the HRP the more opportunities for virus spread may occur. One reason for this comparatively extended HRP could be the delayed reporting of the disease event to the veterinary authorities. Immediate sampling and testing of the first pig showing clinical signs might have revealed disease introduction earlier. Awareness of ASF and facilitated notification might encourage farmers to report suspicious disease events immediately. In the case of ASF, awareness is also of particular importance due to the unspecific clinical signs and the relatively slow spreading. In addition, veterinarians and veterinary technicians should be alerted to the disease to avoid attempts to treat ASFV infected animals. On the presented farm, eleven days passed between the first and the second dead pig. Thus, infected pigs might go unnoticed, as farmers are not connecting disease events to each other. 

### 4.2. Potential Spreading Scenario

The sow (#1) was the first pig that died of ASF on the farm. The other affected pigs died 11–15 days later. This temporal pattern in combination with diagnostic results, allows conclusions regarding the possible spreading of the disease (Figure 2). Only sow (#1) got infected when the virus was introduced into the farm. The pigs (#2–6) that died later most likely did not get infected at that time but later by direct or indirect contact with the infected sow (#1). Animal #7 did not get infected until the culling of all pigs. It was separated from the other pigs only by the service aisle of approximately one-meter width. Direct contact with the animals on the other side of the aisle was impossible but all pigs shared the same food and cleaning equipment. The farmer did not describe a certain cleaning or feeding routine that could explain why pig #7 did not get infected. Direct contact to blood or bloody excretions is described as being the most effective route of disease transmission [20], which could explain the spreading between the pigs on the affected side of the aisle. Lacking of direct contact could explain why the separated animal did not get infected and contributes to the hypothesis of a moderate contagiosity of the disease [21].

### 4.3. Biosecurity and Potential Introduction Pathways

In affected countries, most ASF outbreaks in the domestic pig sector appear in backyard holdings [22]. Due to the mostly low biosecurity level of the farms, the risk of disease introduction is high. At the same time, animals that die are mostly not compensated leading to considerable losses for farmers. This increases the occurrence of emergency selling or slaughter in case of disease suspicion. It contributes to the spread of the disease if potentially contaminated meat, commonly sold for a lower price, is swill-fed in further pig holdings [23]. Despite the fact that swill-feeding is forbidden in the European Union, it is still common practice in small-scale holdings. Moreover, access to small-scale holdings is often hardly limited to the public and animals are moved around for breeding purposes. The definite determination of the introduction route remains often unclear in outbreak investigations, as many hypothetical events are conceivable. However, it is important to find out the most probable introduction routes or to exclude as much as possible and weigh the different risk factors accordingly. In the case of the presented outbreak, anthropogenic factors are seen as the highest risk for disease introduction. Access to animal facilities was not restricted to anyone who had access to the farmer’s house or yard. Thus, direct or indirect contact to contaminated fomites or food, which entered the stable via human activities, is regarded as the most likely source of infection. This anthropogenic factor has been shown to be the most relevant one in the spread of ASF in Eastern Europe [24]. The involvement of wild boar in disease introduction remains unclear. At the time of the outbreak and the following five months there was no evidence for ASF in the local wild boar population despite increased alertness among hunters and the veterinary service. Nonetheless, on 13 February 2019, ASF was detected in a wild boar found dead around 15 km (see Figure 1) away from the farm. It could be speculated that the disease has already been present in the wild boar population since summer 2018 but not detected due to a lack of sampling of fallen wild boar. On the other hand, the disease could have been introduced into the local wild boar population during or after the described backyard outbreak, therefore not qualifying as a source of infection for the domestic pigs affected. Most other sources can be regarded as unlikely (see Table 2).

### 4.4. Applied Control Measures

Disease control under backyard conditions is challenging as many factors have to be taken in consideration and control measures must be adapted to local conditions. Legislation for ASF control and eradication [13] is mainly targeting commercial farms and often ignoring the peculiarities of the backyard sector. In the case of the outbreak in the Bulgarian backyard farm, around half of the households in the affected village owned pigs at a small scale, keeping only one to two animals. Despite the fact that pigs were all kept enclosed, biosecurity of these farms was considered low and the whole village therefore regarded as one epidemiological unit. Consistent with this background, culling of all pigs in the village was implemented as a preventive measure. However, the presented case report shows that an ASF outbreak in the backyard sector can be effectively managed if control measures are adapted reasonably to local conditions and strictly applied. As backyard holdings are often not registered, farmers are on the one hand not easy to identify, on the other hand they may not receive compensations for losses due to control measures. Thus, culling leads to serious financial losses for farmers that should not be underestimated to avoid underreporting of sick animals and disease incidence. If farmers wish to restock their stables, they should be encouraged to register their pig holding. This registration should include the mandatory application of basic biosecurity measures. The uncertain situation of ASF in the local wild boar population emphasizes the importance of passive surveillance. Sampling of fallen wild boar is crucial for early disease detection and effective disease control [25]. Backyard farms feed mainly unprocessed crops from surrounding fields to their pigs that can easily be contaminated by infected wild boar. If they are aware of the disease status in the local wild boar population, further decontamination steps, like heating, could be implemented to prevent this introduction route.

## 5. Conclusions

The presented case can be regarded as an example for ASF in the backyard sector reflecting the diseases dynamics and challenges of disease control. The slow spread of the disease from pig-to-pig contributes to the hypothesis of a moderate contagiosity of ASF, but could hamper early disease detection as it leads to initially low mortalities. The human factor has already been described [21] to be the highest risk factor for disease introduction and spread showing again that awareness of all contributors in the pig value chain is of utmost importance. However, the example demonstrates that—despite the challenging conditions—successful ASF control in the backyard sector is feasible.

## Figures and Tables

**Table 1:** Overview of pigs in the affected farm including test results.

Animal ID	Date of Death	qPCR	Antibody Detection	Virus Isolation	Tested Specimen
**Pregnant sow #1**	Aug 16	positive	n/a	n/a	tissue sample (decomposed)
**Fattening pig #2**	Aug 27	positive	n/a	n/a	tissue sample (decomposed)
**Pregnant sow #3**	Aug 28	positive	n/a	n/a	tissue sample (decomposed)
**Boar #4**	Aug 29	positive	positive	positive	spleen, kidney, lymph node
**Fattening pig #5**	Aug 31 (culled)	positive	weakly positive	positive	blood
**Fattening pig #6**	Aug 31 (culled)	positive	negative	positive	blood
**Fattening pig #7**	Aug 31 (culled)	negative	negative	n/a	blood

**Table 2:** Hypothetical introduction routes and their estimated probability.

Hypothetical Introduction Route	Risk Factors	Description	Probability of Introduction Pathway
1. Contact to contaminated fomites/food	Feeding regime	According to the farmer, no swill was fed to the pigs. No ASF has been detected in other pig farms of the village having the same food supply.	Moderate
	Anthropogenic factor	Access to stable not restricted for anyone who had access to the farmers yard. In case of the farmer’s absence, colleagues were taking care of the animals.	High
2. Link to infected wild boar	Setup of the farm	The animal facilities are located inside the garden surrounding the farmer’s house. The area is enclosed by a stonewalled fence. No evidence for direct contact with wild boar within the farm.	Negligible
	Indirect contact with wild boar environment	The first ASF case in wild boar within the region has been detected 5 months after the described outbreak. Still it cannot be excluded that the disease has been present in the population before then.	Moderate
	Anthropogenic factor	The owner has a hunting license but did not go hunting in the last 15 years.	Low
3. Animal movement	Introduction of infected animals	All pigs have been purchased in 2017.	Negligible
	Animal movement	The last movement of the boar for breeding purposes was three months before the outbreak.	Negligible

## References

[1] Wang T., Sun Y., Qiu H.J. (2018). African swine fever: An unprecedented disaster and challenge to China. Infect. Dis. Poverty.

[2] Penrith M.L., Bastos A.D.S., Etter E., Beltran-Alcrudo D. (2019). Epidemiology of African swine fever in Africa today: Sylvatic cycle versus socio-economic imperatives. Transbound. Emerg. Dis..

[3] Costard S., Wieland B., de Glanville W., Jori F., Rowlands R., Vosloo W., Roger F., Pfeiffer D.U., Dixon L.K. (2009). African swine fever: How can global spread be prevented?. Philos. Trans. R. Soc. B Biol. Sci..

[4] Rowlands R.J., Michaud V., Heath L., Hutchings G., Oura C., Vosloo W., Dwarka R., Onashvili T., Albina E., Dixon L.K. (2008). African swine fever virus isolate, Georgia, 2007. Emerg. Infect. Dis..

[5] World Bank (2007). World Development Report 2008: Agriculture for Development.

[6] Martinez-Lopez B., Alexandrov T., Mur L., Sanchez-Vizcaino F., Sanchez-Vizcaino J.M. (2014). Evaluation of the spatial patterns and risk factors, including backyard pigs, for classical swine fever occurrence in Bulgaria using a Bayesian model. Geospat. Health.

[7] Alexandrov T., Kamenov P., Depner K. (2011). Surveillance and control of classical swine fever in Bulgaria, a country with a high proportion of non-professional pig holdings. Open Agrar.

[8] Chenais E., Sternberg-Lewerin S., Boqvist S., Liu L., LeBlanc N., Aliro T., Masembe C., Ståhl K.J.T.A.H. (2017). African swine fever outbreak on a medium-sized farm in Uganda: Biosecurity breaches and within-farm virus contamination. Trop. Anim. Health Product..

[9] Dione M.M., Akol J., Roesel K., Kungu J., Ouma E.A., Wieland B., Pezo D. (2017). Risk Factors for African Swine Fever in Smallholder Pig Production Systems in Uganda. Transbound. Emerg. Dis..

[10] Bech-Nielsen S., Fernandez J., Martinez-Pereda F., Espinosa J., Perez Bonilla Q., Sanchez-Vizcaino J.M. (1995). A case study of an outbreak of African swine fever in Spain. Br. Vet. J..

[11] Lamberga K., Seržants M., Oļševskis E. (2019). African swine fever outbreak investigations in a large commercial pig farm in Latvia: A case report. Berl. Münch. Tierärztl. Wochenschr..

[12] FAO Regional Office for Europe and Central Asia (2018). FAO Works to Better Understand Backyard Pig Sector, Key in the Fight Against Swine Fever.

[13] Council of the European Union (2002). Council Directive 2002/60/EC of 27 June 2002 Laying Down Specific Provisions for the Control of African Swine Fever and Amending Directive 92/119/EEC as Regards Teschen Disease and African Swine Fever.

[14] Nurmoja I., Motus K., Kristian M., Niine T., Schulz K., Depner K., Viltrop A. (2018). Epidemiological analysis of the 2015–2017 African swine fever outbreaks in Estonia. Prev. Vet. Med..

[15] Gabriel C., Blome S., Malogolovkin A., Parilov S., Kolbasov D., Teifke J.P., Beer M. (2011). Characterization of African swine fever virus Caucasus isolate in European wild boars. Emerg. Infect. Dis..

[16] Nurmoja I., Petrov A., Breidenstein C., Zani L., Forth J.H., Beer M., Kristian M., Viltrop A., Blome S. (2017). Biological characterization of African swine fever virus genotype II strains from north-eastern Estonia in European wild boar. Transbound. Emerg. Dis..

[17] Blome S., Gabriel C., Beer M. (2013). Pathogenesis of African swine fever in domestic pigs and European wild boar. Virus Res..

[18] Hirata D., Doichev V.D., Raichev E.G., Palova N.A., Nakev J.L., Yordanov Y.M., Kaneko Y., Masuda R. (2015). Genetic variation of the East Balkan Swine (Sus scrofa) in Bulgaria, revealed by mitochondrial DNA and Y chromosomal DNA. Anim. Genet..

[19] Forth J.H., Forth L.F., King J., Groza O., Hubner A., Olesen A.S., Hoper D., Dixon L.K., Netherton C.L., Rasmussen T.B. (2019). A Deep-Sequencing Workflow for the Fast and Efficient Generation of High-Quality African Swine Fever Virus Whole-Genome Sequences. Viruses.

[20] Pietschmann J., Guinat C., Beer M., Pronin V., Tauscher K., Petrov A., Keil G., Blome S. (2015). Course and transmission characteristics of oral low-dose infection of domestic pigs and European wild boar with a Caucasian African swine fever virus isolate. Arch. Virol..

[21] Chenais E., Depner K., Guberti V., Dietze K., Viltrop A., Ståhl K. (2019). Epidemiological considerations on African swine fever in Europe 2014–2018. Porc. Health Manag..

[22] Costard S., Mur L., Lubroth J., Sanchez-Vizcaino J.M., Pfeiffer D.U. (2013). Epidemiology of African swine fever virus. Virus Res..

[23] Costard S., Zagmutt F.J., Porphyre T., Pfeiffer D.U. (2015). Small-scale pig farmers’ behavior, silent release of African swine fever virus and consequences for disease spread. Sci. Rep..

[24] Brown V.R., Bevins S.N. (2018). A Review of African Swine Fever and the Potential for Introduction into the United States and the Possibility of Subsequent Establishment in Feral Swine and Native Ticks. Front. Vet. Sci..

[25] Boklund A., Cay B., Depner K., Földi Z., Guberti V., Masiulis M., Miteva A., More S., Olsevskis E., European Food Safety Authority (EFSA) (2018). Epidemiological analyses of African swine fever in the European Union (November 2017 until November 2018). EFSA J..

